# Response to: Comment on “A Better Way to Decrease Knee Swelling in Patients with Knee Osteoarthritis: A Single-Blind Randomised Controlled Trial”

**DOI:** 10.1155/2020/1396539

**Published:** 2020-12-22

**Authors:** Zübeyir Sari, Onur Aydoğdu, İlkşan Demirbüken, S. Ufuk Yurdalan, M. Gülden Polat

**Affiliations:** Marmara University, Faculty of Health Sciences, Dept. of Physiotherapy and Rehabilitation, Istanbul, Turkey

We read with great interest the Letter to the Editor by Broere et al. [1] on our article [2]. We would like to thank the authors for their interest in our study. We have addressed each of their concerns as follows.

In our study, we found significant effects on reducing only knee swelling between the groups. We, therefore, wanted to focus on knee swelling in the introduction part of the study and title for the readers. We did not classify the parameters as primary and secondary outcomes as can be seen in the Methods section. When we registered the protocol, we thought that we had to separate them as primary and secondary outcomes because there was an obligatory section for indicating the secondary outcomes. We do not think that the integrity of the research has been compromised, but we have revised them so that all of the parameters are primary outcomes in the clinical trial protocol registration system as suggested (clinicaltrials.gov).

In the sample size calculation, we used knee joint range of motion because we could not find any published articles about knee swelling as a sample for sample size calculation. The manuscript was originally submitted without a sample size calculation, and reviewers asked us to include one with a reference. We, therefore, calculated the sample size with an article about one of the parameters which was knee joint range of motion.

We believe that calculating the sample size with knee joint range of motion in addition to focusing more on one of the parameters (knee swelling) in introduction part and title of the study, does not compromise the research integrity.

In the original submission, the following paragraph was included:

“Osteoarthritis (OA), which is one of the most common rheumatological conditions, is a degenerative joint disease. OA occurs most commonly in the knee. Individuals with knee OA suffer progressive loss of function and display increased dependency in walking, stair climbing, and other tasks involving lower extremities. Advanced functional loss, problems in standing, ascending and descending stairs, balance, and gait are common in patients with knee OA, accompanied by pain, joint stiffness, and reduced quality of life.

One of the most common symptoms of knee OA is swelling. Knee swelling negatively affects knee mechanics and muscle activity in patients with OA.”

We removed this paragraph on the advice of the reviewers, who suggested to omit this paragraph because it was considered to be well established and not essential to the manuscript. However, there is a statement in the introduction part of the study that knee swelling is the most common symptom of knee osteoarthritis in our study, which remained after we removed the original paragraph: “Swelling is the most common symptom of knee osteoarthritis (OA), which negatively affects knee mechanics and muscle activity in patients with OA.” This may have occurred because our native language is not English. We thought that we might emphasize knee swelling because it was the only significant parameter in the study. In addition, it is known that knee swelling and pain are factors that affect functional recovery and patient satisfaction greatly in many conditions including knee osteoarthritis [3]. We can show that knee swelling—including the terms effusion and edema—is one of the most common symptoms of knee osteoarthritis in published articles; however, it is not the most common symptom [4].

The letter also comments on our trial registration protocol. Before we submitted the manuscript, we received approval from the Editorial Office regarding whether we could create a new registration number after finishing the enrollment of the patients. In addition, there are many published studies [5, 6] whose trial protocol was registered several years after completion of data collection. It may be correct that earlier registration is better; however, this was the first time that we had registered one of our studies to the protocol registration system. We have now learned this system for the future.

The letter refers to participant characteristics and dropouts. There were 9 dropouts in one group. We had heard about “intention-to-treat analysis”; however, we did not have the full details about it because it is not common. We had two existing groups; IPC group (*n* = 45) and cold-pack group (*n* = 36). We formed a new, third group (*n* = 45) which includes the 9 dropouts in addition to the cold-pack group (*n* = 36) to make the “intention-to-treat analysis.” As suggested by Broere et al., after the “intention-to-treat analysis” was formed, we compared the pretreatment values in the IPC group (*n* = 45), cold-pack group (*n* = 36), and the new group (*n* = 45). We found that there are no significant differences (*p* > 0.05) between the new, third group and the first two groups. According to these results, exclusion or inclusion of 9 dropouts is not expected to affect the results. We are also thankful for the suggestion.

The inclusion criteria were also commented on in the letter. All patients who were diagnosed by the doctor as having knee osteoarthritis according to the criteria of the American College of Rheumatology (ACR) were participants in the study. In addition, we can show many published studies which have same inclusion criteria [6, 7]. The patients who were diagnosed as either OA stage 2 or 3 (severity) according to the Kellgren–Lawrence criteria were included in the study; however, we did not examine and classify the patients according to their knee compartments. We thank the reader for this suggestion but also note that this is a difficult assessment method for use in clinical settings.

Finally, the letter referred to the use of measuring tape to quantify knee joint swelling. We believe that all of our parameters are validated and widely accepted methods, including use of a tape measure for knee joint swelling. We may say that the tape measure is a reliable method for the determination of gross changes in knee swelling in patients [8]. However, we can also accept that the parameters which we used in the study are not very objective methods. In the Discussion section, we mentioned that there were some limitations to this study. The most significant limitation of this study was that we assessed knee swelling using a tape measure. A more objective evaluation including the use of imaging techniques would be better. The authors recognize the criticism and limitation to the study but also note that, because the clinic had limited opportunities 10 years ago, we could not access such objective measurement methods. In addition, unfortunately, we cannot comment on this outcome measure's reliability. It is correct that we interpreted the results according to only *p* values. It would be better to interpret the results according to effect sizes and uncertainty metrics in addition to *p* values in line with current best practice. We, therefore, added the effect sizes in the following table. To evaluate the effect size, Cohen's *d* coefficient was calculated for between-group variables. An effect size of 0.20 to <0.50 was regarded as small, 0.50 to <0.80 as medium, and >0.80 as large [9, 10] (Table 1).

In summary, we thank the letter authors and the editor for the chance to clarify the details of our study to the readers.

## Figures and Tables

**Table 1:** Comparison of effect size values of the change in the outcome measures for the IPC and cold-pack groups.

Outcomes	IPC group (*n* = 45)	Cold-pack group (*n* = 45)	*t*	*p*	Effect size (ES)
∆Knee flexion (degree)	6.33 ± 6.51	6.25 ± 7.96	−0.008	0.994	<0.50
∆Quadriceps MS (N/m)	6.53 ± 10.72	4.28 ± 6.73	1.262	0.211	<0.50
∆Hamstring MS (N/m)	4.02 ± 1.62	2.68 ± 1.45	1.825	0.072	>0.80
∆Knee swelling (cm)	−2.60 ± 0.86	−0.49 ± 0.92	2.243	0.028^*∗*^	>0.80
∆Pain intensity (cm)	−1.78 ± 1.80	−1.46 ± 1.74	−1.481	0.143	<0.50
∆WOMAC-pain (score)	−1.91 ± 2.43	−1.80 ± 2.03	0.120	0.905	<0.50
∆WOMAC-stiffness (score)	−0.28 ± 1.25	−0.11 ± 1.40	−0.716	0.476	<0.50
∆WOMAC-physical function (score)	−3.91 ± 5.91	−4.13 ± 4.51	−0.237	0.813	<0.50
